# Infant Social Attention Associated with Elevated Likelihood for Autism Spectrum Disorder: A Multi-Method Comparison

**DOI:** 10.1007/s10803-024-06360-z

**Published:** 2024-04-28

**Authors:** Xiaoxue Fu, Emma Platt, Frederick Shic, Jessica Bradshaw

**Affiliations:** 1 Department of Psychology, University of South Carolina, Columbia, SC USA; 2 Center for Child Health, Behavior and Development, Seattle Children’s Research Institute, Seattle, WA USA; 3 Department of Pediatrics, University of Washington School of Medicine, Seattle, WA USA

**Keywords:** Eye Tracking, Manual Coding, Social Attention, Infant, Autism

## Abstract

**Purpose**: The study aimed to compare eye tracking (ET) and manual coding (MC) measures of attention to social and nonsocial information in infants with elevated familial likelihood (EL) of autism spectrum disorder (ASD) and low likelihood of ASD (LL). ET provides a temporally and spatially sensitive tool for measuring gaze allocation. Existing evidence suggests that ET is a promising tool for detecting distinct social attention patterns that may serve as a biomarker for ASD. However, ET is prone to data loss, especially in young EL infants. **Methods**: To increase evidence for ET as a viable tool for capturing atypical social attention in EL infants, the current prospective, longitudinal study obtained ET and MC measures of social and nonsocial attention in 25 EL and 47 LL infants at several time points between 3 and 24 months of age. **Results**: ET data was obtained with a satisfactory success rate of 95.83%, albeit with a higher degree of data loss compared to MC. Infant age and ASD likelihood status did not impact the extent of ET or MC data loss. There was a significant positive association between the ET and MC measures of attention, and separate analyses of attention using ET and AC measures yielded comparable findings. These analyses indicated group differences (EL vs. LL) in age-related change in attention to social vs. nonsocial information. **Conclusion**: Together, the findings support infant ET as a promising approach for identifying very early markers associated with ASD likelihood.

## Introduction

Infant attention plays a central role in cognitive and socioemotional development. Looking behavior provides a window into infant perception and cognition (Aslin, 2007; Oakes, 2010). Studying social attention by measuring looking behavior toward social and nonsocial stimuli during screen-based tasks informs key mechanisms implicated in the early development of autism spectrum disorder (ASD) (Chita-Tegmark, 2016; Falck-Ytter et al., 2022). Manual coding (MC) of looking behavior by trained researchers is an accessible and well-established method for measuring cognitive capacities such as visual detection, preferences, discrimination, expectations, and learning in infants who cannot make verbal or manual responses (Aslin, 2007, 2012). However, MC is a relatively subjective, indirect, and laborious method for measuring the macrostructure of looking behavior (Aslin, 2007, 2012). Relative to MC, eye tracking (ET) tends to have high temporal and spatial resolution that enables direct assessment of the microstructure of looking behavior (Aslin, 2012; Wass et al., 2013).

ET studies have revealed that ASD is characterized by reduced social attention, a promising biomarker for ASD (e.g., Shic et al., 2022; Wen et al., 2022). However, infant ET is prone to reduced data quality, particularly in children with ASD (Hessels & Hooge, 2019; Venker & Kover, 2015; Wass et al., 2014). This compromised data quality can negatively impact ET data reliability and validity (Hessels & Hooge, 2019; Wass et al., 2014) and impede accurate identification of prodromal and acute markers of ASD. The goal of the present study is to evaluate the utility of ET for measuring social attention in infants with elevated familial likelihood of ASD (EL) and low familial likelihood of ASD (LL). It is estimated that 10–20% of younger siblings of children with ASD go on to receive an ASD diagnosis themselves (Constantino et al., 2010; Ozonoff et al., 2011). Studying young infant siblings of children with a confirmed ASD diagnosis facilitates the detection of precursors for later clinical diagnosis before the risk markers are confounded by the development of compensatory mechanisms (Elsabbagh & Johnson, 2010). Infant sibling design can also reveal sub-clinical traits and characteristics associated with broader autism phenotype (Elsabbagh & Johnson, 2010; Rogers, 2009). We aim to (1) characterize and compare measures of attention, inattention, and data loss that are derived from ET and MC measures of attention during a screen-based task; (2) investigate participant-specific factors that influence the availability of ET and MC data; (3) examine the association of ET and MC measures of attention; and (4) compare ET and MC attention results to determine whether both measures indicate group differences (EL versus LL) in developmental changes of social attention. Findings can inform infant ET practices, contribute to evidence that validates the use of ET for identifying early-emerging risk markers and help researchers make informed decisions on whether and when to use MC in the face of ET data loss.

Individual differences in the tendency to attend to social information emerge in early infancy. ET free-viewing studies show that typically developing LL infants (between 3 and 30 months) exhibit more face looking as they become older (Frank et al., 2009, 2012). Furthermore, older infants are more capable of adaptively allocating their attention to locations in complex social scenes that help to extract social information, such as mouth looking when the actor was talking (Frank et al., 2012). In contrast, the Autism Biomarkers Consortium for Clinical Trials study (ABC-CT; McPartland et al., 2020) in 6-11-year-old children (*N* = 280) revealed that children with ASD spend less time looking at faces across three ET tasks presenting social scenes (Shic et al., 2022). This ET measure of face looking met established criteria for biomarker viability (McPartland et al., 2020) and is being further evaluated as an FDA-approved biomarker for ASD in school-age children (Shic et al., 2022).

Identifying potential ET biomarkers before age three, when a reliable ASD diagnosis can be determined, is of critical importance for early diagnosis and treatment (Bradshaw et al., 2019; Frazier et al., 2021). For example, Wen et al. (2022) implemented a preferential looking paradigm (Pierce et al., 2011, 2016) that displays a social and nonsocial video side-by-side in infants and toddlers (12 to 48 months; *N* = 1685). They found that toddlers with ASD spent the highest proportion of time looking at the dynamic geometric images (relative to dynamic social images) compared to their peers with ASD features, developmental delays, or typically developing children. The ET measure had satisfactory classification accuracy in distinguishing ASD from non-ASD children.

Furthermore, ET’s relatively high spatial and temporal resolution enables researchers to identify nuanced differences in social attention patterns between EL (with or without later ASD diagnosis) and typical developing LL infants. For example, using smaller areas of interest (AOIs), six-month-old EL infants were found to spend less time looking at the eyes, nose, and mouth than LL infants when viewing dynamic talking faces (Shic et al., 2014). Moreover, in a task that presented a face embedded within an array of distractors, EL infants displayed “sticky attention” characterized by periods with prolonged fixations, attended to fewer AOIs, and were more likely to return to previously fixated areas in the visual array (Elsabbagh et al., 2013; Gliga et al., 2018; Hendry et al., 2018). Hence, compared to LL infants who gain more efficiency in allocating their attention based on social context (Frank et al., 2012), ET studies have shown that elevated likelihood for ASD might be associated with less adaptive attention allocation to social information.

The critical importance of identifying ET biomarkers in infants and toddlers highlights the need to evaluate challenges in collecting good quality ET data in this population. For screen-based ET methods, participants’ corneal reflection and pupil locations need to be detected to determine the point of gaze (POG) with high validity and reliability (Wass et al., 2014). ET data quality can be characterized by accuracy and robustness. Low accuracy refers to a large deviation between the POG reported by the eye tracker and the actual POG. To obtain accurate data, participants must look at a set of targets on screen during the calibration session which is necessary for determining the POG on the screen for each recorded sample (Oakes, 2012). Low robustness is characterized by fragmented ET recording streams, such that data “flicker off” when the eye tracker cannot detect the corneal reflection or pupil (Wass et al., 2014). Compared to adults, infants have lower compliance in looking at the calibration and validation targets, poorer head control, and greater head and body movement, are more likely to get fussy, and often have droopy and watery eyes that can obstruct corneal reflection and pupil detections. These infant characteristics can lead to lower ET data accuracy and robustness even in infants who completed the ET tasks (Hessels et al., 2015; Wass et al., 2013, 2014).

ET data loss and ET data quality may bias the results and interpretations. Participant exclusion rates in ET studies with EL and/or LL infants and toddlers can reach above 30% (e.g., Chawarska et al., 2016; Chawarska et al., 2013; Pomaranski et al., 2021). Hence, insufficient sample sizes can yield false positive or negative results, and the findings might not be representative of infants who could not provide sufficient ET data (Oakes, 2017). Furthermore, systematic differences in ET data quality can create confounding influences on ET dependent variables of interest, thus bias interpretations (Hessels & Hooge, 2019; Wass et al., 2014). Age and ASD status impact ET data quality. Among typically developing children (18 months to 11 years of age), younger age is related to lower accuracy (Dalrymple et al., 2018). EL infants (6–10 months) provided marginally less robust data (i.e., more ET data “flicker off”) than LL infants (Wass et al., 2015). Consistent with this finding, ASD symptoms in children were related to lower data quality, indexed by a lower percentage of trackable data and reduced accuracy (Shic et al., 2022; Wang et al., 2023). Poor accuracy reduces validity in determining the location of POGs and the spatial resolution in interpretations about whether or not the infant is looking at a particular area of interest (Aslin, 2012). Low robustness is related to shorter fixation durations (Wass et al., 2013, 2014, 2015). Hence, systematic individual differences in ET data quality may limit the validity and reliability of key metrics of looking behavior (Hessels & Hooge, 2019; Wass et al., 2013).

There are strategies that researchers can take to reduce the potential bias caused by problematic ET data quality, especially for populations who may already be prone to low data quality. These include conducting MC to confirm ET findings (Venker et al., 2020; Wass et al., 2013). In manual coding, trained human coders determine whether the POG falls within the predefined AOIs in predefined time frames (e.g., frame-by-frame or second-by-second). MC of looking behavior using large-AOI tasks (Teller, 1979) is a well-established method that has provided an important understanding of infant development of attentional functions (Oakes & Amso, 2018), including social attention (Kinzler et al., 2007; Peltola et al., 2008).

Relative to ET, MC is robust against data rejections due to low quality data in infant studies where collecting sufficient data is challenging. MC only requires clear video images of participants’ heads and eyes, as opposed to the detection of corneal reflection and pupil required for ET. Less robust ET data leads to under-estimations of looking time in ET relative to MC data (Wass et al., 2013). When a given research question does not demand characterizing the microstructure of looking behavior, MC may provide an effective method for accessing infant looking behavior (Aslin, 2012). The advantage of MC might be more significant for children with ASD who may be more susceptible to low ET data quality (Shic et al., 2022; Venker & Kover, 2015). Venker et al. (2020) conducted a within-subject comparison of MC and ET assessment using the same task in toddlers with ASD (24 to 36 months). They found that more toddlers provided usable MC than ET data, and MC yielded more usable trials than ET data. Moreover, the task-condition effect was significant only for MC but not ET data. It is possible that, with more data availability (e.g., more usable trials per participant), MC might provide a better representation of participants’ true looking behavior (Venker et al., 2020).

The present study leverages longitudinal data collected in the first two years of life to examine developmental changes in looking behavior toward social and nonsocial information in LL and EL infants using both ET and MC data collected from a free-viewing task. Three measures are used to compare ET and MC data collection methods: % screen looking, % off-screen looking, and % data loss. Attention was indexed by the percentage of time looking at the presentation screen (% screen looking). Inattention was indexed by the percentage of time looking away from the screen (% off-screen looking). ET data loss at the visit level was due to unsuccessful calibration. MC data for these visits were available. At the trial level, ET data loss was computed as the percentage of time during which the location of the POG could not be estimated (ET % data loss), and MC data loss was computed as the percentage of time during which the trained coder could not determine the gaze location (MC % data loss). For both ET and MC, the percentage of valid data (% valid data = % screen looking + % off-screen looking) was also computed.

We first examine ET and MC measures of % screen looking, % off-screen looking, and % data loss. We then examine differences in overall % valid data between ET and MC. We hypothesized that ET would yield lower % valid data compared to MC. Second, we investigated the effect of infant age and familial likelihood (LL versus EL) on % valid data for ET and MC, separately. We hypothesized that, for ET, lower infant age will be associated with lower % valid data, and EL infants will have reduced % valid data compared to LL infants. We also anticipated that the effect of age and familial likelihood will not be significant for MC % valid data. Third, we examined the association between ET and MC % screen looking. It was hypothesized that there will be a significant ET-MC association in % screen looking. Lastly, we examined the impact of age and familial likelihood on % screen looking toward the social and nonsocial stimuli in ET and MC data separately, and we inspected whether ET and MC yield comparable findings. We expected that both ET and MC would show group differences between EL and LL infants in developmental changes in % screen looking toward social and nonsocial information. Our findings can inform participant-level factors that may influence ET data loss, and whether both ET and MC measures of the macrostructure of looking behavior can reveal early markers of ASD likelihood.

## Methods

### Participants

Participants were part of a longitudinal study on social development and early detection of autism spectrum disorder (ASD). Participants included 72 infants (34 females) seen longitudinally from 3 to 24 months of age. Eligibility criteria for all participants included: enrollment into the study prior to 6 months of age, full-term birth (≥ 37 weeks gestation), completion of ET task during at least one visit, no congenital vision or hearing abnormalities, and no known genetic syndromes (e.g., Down syndrome and Fragile-X syndrome). Among the participating infants, *N* = 25 infants (13 females; *M*_age_=10.89 months, *SD* = 6.29) were at an elevated familial likelihood of ASD, defined as having a full biological sibling with a confirmed diagnosis of ASD. The race breakdown for EL infants was: 0% Asian, 16% African American, 68% white, and 16% mixed race. Moreover, 12% of EL infants were Hispanic. Forty-seven infants (21 females; *M*_age_=11.24 months, *SD* = 6.59) were at low familial likelihood of ASD and had no first- or second-degree relatives with ASD. The race breakdown for LL infants was: 4% Asian, 32% African American, 55% white, and 9% mixed race. One (2%) of the LL infants was Hispanic. Infants were invited to the laboratory at 3, 4, 6, 9, 12, 15, 19, and 24 months, with a total of 216 visits completed. All procedures were approved by the University Institutional Review Board and families completed informed consent prior to the study procedures.

### Task Conditions

**Fig. 1:**
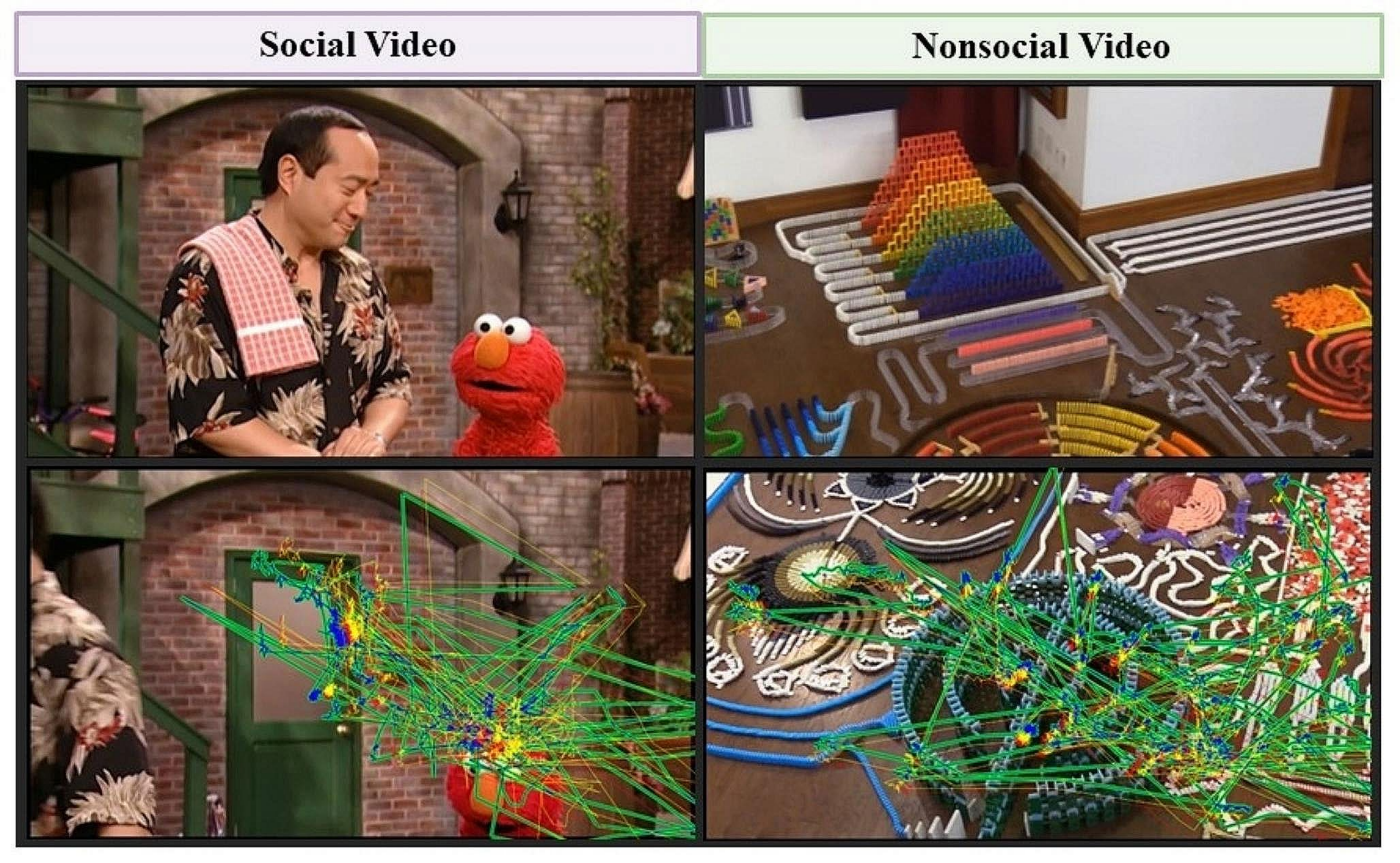
Examples of the Social and Nonsocial video stimuli. The task consisted of a Social condition and a Nonsocial condition, with the order counterbalanced across participants. Each task condition consisted of 5 trials, each presented a dynamic video clip for 36 to 93 s, with a total duration of 4 min and 55 s

The screen-based attention task examines attention across two conditions: Social and Nonsocial videos (Fig. 1). Each task condition contained 5 trials and was presented sequentially such that participants saw one entire task condition (e.g., 5 Social videos) before the other task condition (e.g., 5 Nonsocial videos). The order of Social and Nonsocial condition presentations was counterbalanced across participants. Each trial presented a dynamic video clip for 36 to 93 s, with a total duration of 4 min and 55 s for the block. The Social videos presented two animate agents (puppets or people) interacting and communicating with each other. The Social videos included select scenes from Baby Einstein (2 trials) and Sesame Streat (1 trial), as well as lab-generated videos of a child and a mother playing with toys and communicating (2 trials). The Nonsocial videos were designed to match the Social videos in terms of visual (animation versus live content) and auditory (with or without music and audio-visual synchrony) components. The Nonsocial videos showed animated fractals with classical music (2 trials) and chain reaction / cause-and-effect scenes with objects (2 trials) and colorful dominos (1 trial). None of the Nonsocial videos contained people, faces, language, or other social or social-like agents.

### ET Data Acquisition and Processing

The ET data were acquired during the task presentation at a 60 Hz sampling rate using an SR Research Eyelink 1000 eye tracker (SR Research Ltd.) with the monocular remote desktop configuration. Stimuli were presented on a 21-inch (1680 × 1050 pixels) monitor and controlled via Presentation (Version 23.0, Neurobehavioral Systems, Inc., Berkeley, CA, www.neurobs.com). During the task, infants sat on a car seat at a 95 cm viewing distance from the stimulus presentation monitor. A 5-point calibration procedure was presented at the beginning of the task during which an audiovisual animation was displayed at the center and four corners of the screen. A 5-point validation procedure was presented at the end of each block to facilitate error estimation and scanpath recalibration (Shic et al., 2022). Data collection continued until all trials have been presented, or the infant has stopped attending the presentation. ET data were processed using a pipeline written in MATLAB and Perl (Shic, 2008; Shic et al., 2022). The pipeline incorporated calibration and area-of-interest (AOI) fixation computations. The AOI for the current analysis is the entire screen. For each trial, we computed the percentage of time (over the trial duration) looking at the screen AOI (% screen looking), the percentage of time looking off-screen (% off-screen looking), and the percentage of time during which the location of the POG could not be determined (% data loss). In addition, % valid data was calculated as the sum of % screen looking and % off-screen looking (% valid data = % screen looking + % off-screen looking). The internal consistency of the ET % screen looking measure was estimated using a permutation-based split-half approach with 5000 random splits of the trials (*splithalf* R package). Table 1 provides the Spearman-Brown corrected split-half reliability (Sears et al., 2019) scores for the % screen looking by age of assessment.

**Table 1:** Internal consistency and data loss by age of assessment. Internal consistency is indexed by Spearman-Brown corrected split-half reliability scores for eye-tracking (ET) and manual coding (MC) measure of the percentage of screen looking time relative to the stimulus presentation time

	Internal Consistency	
	ET	MC
3	0.89*	0.83*
4	0.91*	0.90*
6	0.78*	0.77*
9	0.80*	0.73*
12	0.94*	0.85*
15	0.88*	0.73*
18	0.72*	0.64*
24	0.78*	0.87*

*Note*: * marks significant correlation indicated by the 95% confidence interval; ET = eye tracking; MC = manual coding

### Manual Coding (MC) of Attention

Trained research assistants (RAs) coded infant looking towards the screen during task stimulus presentation. Coding was performed frame-by-frame (30 Hz). For each frame, coders determined whether the infant was looking at the screen (“screen looking”), the infant was looking away from the screen (“off screen looking”), or the infant looking could not be determined (“data loss”). Screen looking was determined if the eyes were visible and open while the head and/or pupil were turned towards the screen. Off-screen looking was determined if the eyes were visible and open while the head and/or pupil were turned away from the screen. Data loss was determined if eyes were not visible or eyes were closed, typically due to camera obstruction (e.g., hand in front of eyes), infant crying, or infant sleeping. The RAs also coded the onset and offset for screen looking, off-screen looking, and data loss. To ensure interrater reliability, 13% of visits were double coded by a second coder (*κ* = 0.79). As with ET data, we calculated trial-by-trial % screen looking, % off-screen looking, and % data loss. In addition, as with ET data, % valid data was calculated as the sum of % screen looking and % off-screen looking (% valid data = % screen looking + % off-screen looking). Table 1 provides the Spearman-Brown corrected split-half reliability scores for % screen looking by age of assessment.

### Data Analysis

Data analyses are performed using R (R Core Team, 2021). First, descriptive statistics are computed for % screen looking, % off-screen looking, and % data loss by method (ET, MC) and age of assessment. The descriptive statistics are presented in Table 2. Second, to test whether ET provided lower % valid data than MC, a mixed effects (LME) model (*nlme*: Pinheiro et al., 2022) was fitted to examine the effect of method (ET versus MC) on % valid data, controlling for the effect of infant age and familial likelihood (LL versus EL). Third, we examined whether infant age and familial likelihood influenced % valid data for ET and MC. Two LME models were fitted to examine the effect of age, familial likelihood, and their interaction effect on % valid data computed from the ET and MC measure, respectively. The second and third analyses included data from visits where either ET, MC, or both data types were available. Fourth, we examined the association between ET and MC measures of % screen looking, controlling for the effect of age and familial likelihood. An LME model was fitted with age, familial likelihood, and ET % screen looking as fixed effects. The outcome of the model was MC % screen looking. ET % screen looking was also entered as a random slope to allow the effect of ET % screen looking to vary across participants. The analysis of ET-MC association included data from visits where both ET and MC data were available. Lastly, we investigated whether the ET and MC measure show comparable results for the three-way interaction effect between age, familial likelihood, and task condition (Social versus Nonsocial videos) on % screen looking. Two LME models were fitted using the ET % screen looking and MC % screen looking as the outcome variable, respectively. Age, familial likelihood, task condition (Social versus Nonsocial videos), and their interaction effects were entered as fixed effects for both models. The main analyses used trials where the infant provided both ET and MC data (Venker et al., 2020). There were a total of 1923 ET and MC trials, respectively. We also tested the effects of age, familial likelihood, and task condition (Social versus Nonsocial videos) on % screen looking with all available ET (*n* = 1932) and MC trials (*n* = 2007), and the results are presented in the Supplemental Information (SI). The parameter estimates and confidence intervals for all models tested in the main analyses are presented in Table 3.

**Table 2:** Descriptive Statistics. Participant number, mean and standard deviation for % screen looking, % off-screen looking, and % data loss measures obtained from eye tracking (ET) and manual coding (MC) by age of assessment. Each measure was averaged across trials and then averaged across participants for a given age of assessment

Age of Assessment (Months)	Participant N		Mean % Screen Looking (SD)		Mean % Off-Screen Looking (SD)		Mean % Data Loss (SD)	
	ET	MC	ET	MC	ET	MC	ET	MC
3	17	19	63.50(19.55)	82.53(15.29)	10.29(13.14)	13.84(13.36)	26.21(14.56)	2.90(6.48)
4	28	28	73.36(17.59)	84.35(17.33)	5.70(8.83)	13.51(17.32)	20.94(16.08)	1.68(3.39)
6	37	39	68.78(16.90)	77.80(18.15)	4.64(3.75)	16.12(12.97)	26.58(15.90)	5.31(12.89)
9	28	31	66.75(22.19)	76.07(23.51)	3.39(2.39)	14.48(12.22)	29.85(22.18)	4.49(12.75)
12	29	30	66.93(23.54)	78.90(20.74)	2.08(1.40)	14.61(13.20)	30.99(23.30)	3.73(7.90)
15	22	23	67.51(19.80)	84.60(11.82)	3.52(4.59)	13.39(10.85)	28.98(18.38)	1.47(3.76)
18	25	25	67.41(13.53)	83.07(10.21)	2.65(4.05)	15.26(9.85)	29.93(13.35)	1.28(3.11)
24	21	21	72.02(14.11)	85.88(9.93)	1.45(1.27)	12.71(7.96)	26.53(14.18)	1.28(4.65)

**Table 3:** Model estimates and confidence intervals

Parameters	Estimate	95% CI	R^2^
**Analysis 1 outcome: mean % valid looking**			
**Fixed effects**			0.39
Intercept	**82.57**	(80.32, 84.82)	
Age	0.07	(-0.16, 0.30)	
Group	1.85	(-0.43, 4.14)	
Method (ET vs. MC)	**-11.60**	(-12.83, -10.37)	
**Random effects**			
SD Intercept	7.10	(5.38, 9.36)	
Residual	12.84	(11.93, 13.82)	
**Analysis 2a outcome: mean ET % valid looking**			
**Fixed effects**			0.03
Intercept	**70.13**	(66.61, 73.63)	
Age	-0.12	(-0.53, 0.28)	
Group	2.92	(-0.62, 6.46)	
Age × Group	0.12	(-0.28, 0.52)	
**Random effects**			
SD Intercept	10.77	(7.80, 14.86)	
Residual	14.15	(12.51, 16.00)	
**Analysis 2b outcome: mean MC % valid looking**			
**Fixed effects**			0.02
Intercept	**95.04**	(93.35, 96.73)	
Age	0.18	(-0.07, 0.43)	
Group	1.20	(-0.51, 2.91)	
Age × Group	-0.01	(-0.26, 0.24)	
**Random effects**			
SD Intercept	2.73	(0.83, 8.95)	
Residual	10.60	(9.45, 11.90)	
**Analysis 3 outcome: mean MC % screen looking**			
**Fixed effects**			0.34
Intercept	**48.52**	(42.10, 54.95)	
Age	**0.19**	(0.05, 0.33)	
Group	-0.16	(-1.84, 1.52)	
ET % screen looking	**0.50**	(0.43, 0.58)	
**Random effects**			
SD Intercept	24.78	(20.41, 30.07)	
ET % screen looking	0.29	(0.24, 0.36)	
Residual	14.87	(14.39, 15.37)	
**Analysis 4a outcome: mean ET % screen looking**			
**Fixed effects**			0.03
Intercept	**67.01**	(63.70, 70.32)	
Age	0.16	(-0.25, 0.56)	
Group	2.90	(-0.47, 6.27)	
Condition	**1.96**	(1.00, 2.92)	
Age × Group	-0.02	(-0.43, 0.39)	
Age × Condition	0.12	(-0.03, 0.26)	
Group × Condition	**1.00**	(0.04, 1.97)	
Age × Group × Condition	**0.21**	(0.06, 0.35)	
**Random effects**			
SD Intercept	9.30	(6.32, 13.69)	
Residual	19.64	(18.99, 20.31)	
**Analysis 4b outcome: mean MC % screen looking**			
**Fixed effects**			0.03
Intercept	**81.48**	(79.07, 83.90)	
Age	0.14	(-0.21, 0.49)	
Group	2.32	(-0.14, 4.78)	
Condition	**1.70**	(0.88, 2.53)	
Age × Group	-0.02	(-0.37, 0.33)	
Age × Condition	**0.20**	(0.07, 0.32)	
Group × Condition	**0.92**	(0.10, 1.75)	
Age × Group × Condition	**0.14**	(0.02, 0.27)	
**Random effects**			
SD Intercept	4.13	(1.48, 11.53)	
Residual	16.84	(16.29, 17.42)	

*Notes*: Beta estimates are bolded if their 95% CI does not contain zero, suggesting a significant effect. *p* values are reported in text. ET = eye tracking; MC = manual coding

## Results

### Comparison Between ET and MC Measurements

ET data was not obtained for 9 visits (4.17% of all visits) where 9 infants (4 EL infants; 7 males; *M*_age_=8.21 months, *SD* = 3.93) were not able to successfully complete the calibration session. There was no group difference between infants who provided ET data (with satisfactory calibration) and those who did not, *p* = .53. However, the MC data were obtained for these visits. Table 2 provides descriptive statistics for % screen looking, % off-screen, and % data loss for ET and MC data by age of visit. It indicates that ET yielded a lower % screen looking, lower % off-screen looking, and greater % data loss compared to MC across all ages of assessment.

Next, we examined the between-method difference in % valid data (i.e., % screen looking + % off-screen). The LME model revealed a main effect of method, *F*(1, 349) = 343.85, *p* < .001, controlling for the effect of infant age, *p* = .55, and group, *p* = .11. ET yielded lower % valid data (i.e., more % data loss) relative to MC, *B*=-11.60, *SE* = 0.63, *t*=-18.46, 95% *CI*[-12,83, -10.37], *p* < .001 (Table 3).

### The Effect of Age and Group on ET and MC % Valid Data

There was no significant effect of infant age, *p* = .55, group, *p* = .10, or age-by-group interaction effect, *p* = .56 on ET % valid data. Similarly, age, *p* = .15, group, *p* = .16, and their interaction effect, *p* = .92, were also not significant on MC % valid data (Table 3).

### The Association Between ET and MC % Screen Looking

**Fig. 2:**
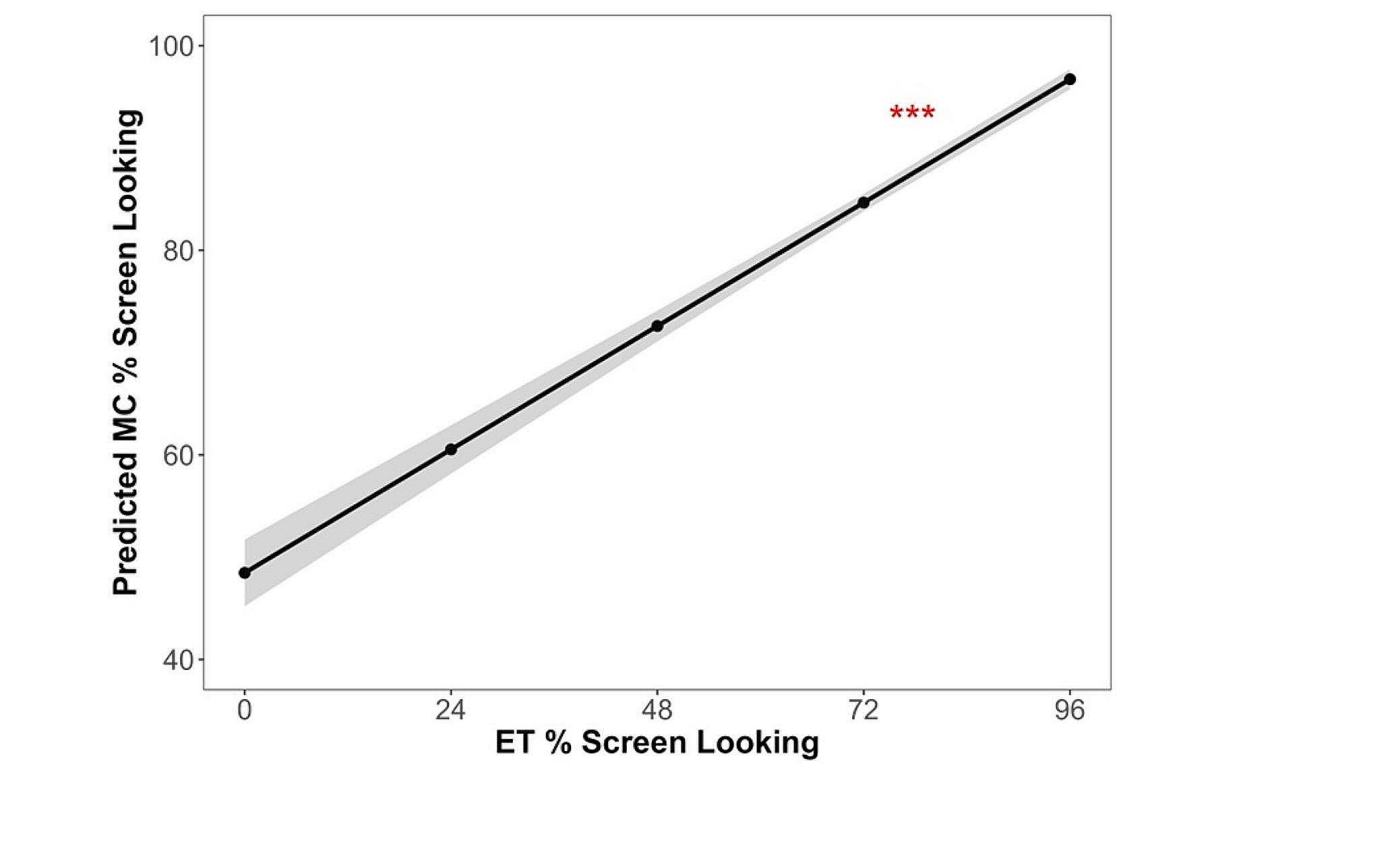
The association between eye tracking (ET) and manual coding (MC) measures the proportion of time looking at the screen over the stimulus presentation duration (% screen looking). Infant age and likelihood (i.e., group: low likelihood versus elevated likelihood) were entered as a covariate in the model *Notes*: ***p < .001

We examined the relation between ET and MC measures of % screen looking, controlling for the effect of age and group. The analysis included data from the visits where both ET and MC data were available. That is, the MC measures of % screen looking were excluded for the 9 visits where ET data were not collected due to unsuccessful calibration. We found that the ET measure of % screen looking was positively related to the MC measure of % screen looking across trials, *F*(1, 1850) = 164.69, *p* < .001. The association was significant over and above the effect of age, *F*(1, 1850) = 7.07, *p* = .008, and group, *p* = .85. The ET-MC association of % screen looking is displayed in Fig. 2, and the model parameter estimates are provided in Table 3.

### The Effect of Age, Group, and Task Condition on ET and MC % Screen Looking

**Fig. 3:**
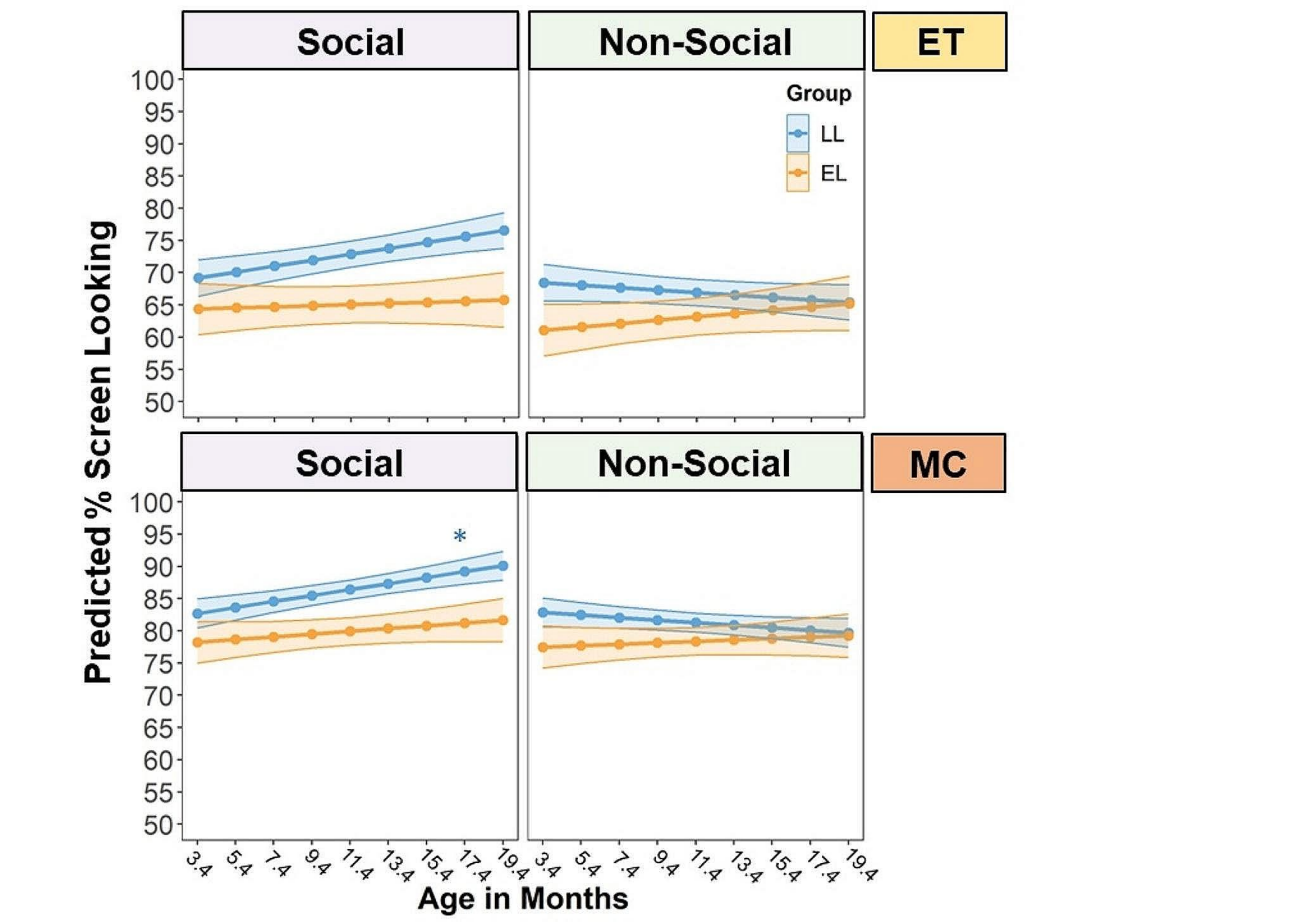
The interaction effect of age, autism likelihood (i.e., group: low likelihood versus elevated likelihood) and condition (Social versus Nonsocial) on the proportion of time looking at the screen over the stimulus presentation duration (% screen looking), separately for the eye tracking (ET) and manual coding (MC) measures. Data of trials with both ET and MC data were included in model testing. Age was mean-centered for model fitting (*M* = 11.4) *Notes*: *p < .05; LL = low likelihood; EL = elevated likelihood

We first examined the age-related changes in ET % screen looking during the Social and Nonsocial condition in the LL and EL infants (Table 3). The LME model revealed a significant effect of condition, *F*(1,1712) = 15.92, *p* < .001, and a significant group-by-condition effect, *F*(1,1712) = 4.17, *p* = .04, which was modified by a significant three-way interaction between age, group, and condition, *F*(1,1712) = 7.73, *p* = .006. The upper panel of Fig. 3 displays the interaction effect on the ET % screen looking. The LL infants displayed a greater % screen looking toward the Social relative to the Nonsocial videos as they grew older, *B* = 0.32, *SE* = 0.08, *t* = 3.97, 95% *CI*[0.16, 0.49], *p* < .001. In contrast, the EL infants did not show developmental changes in % screen looking to the Social versus Nonsocial videos, *p* = .49. The group difference was also manifested in higher % screen looking in LL compared to EL infants for Social stimuli across age, *B* = 3.90, *SE* = 1.76, *t* = 2.22, 95% *CI*[0.40, 7.40], *p* = .03.

We then tested the effect of age, group, and task condition on the MC % screen looking (Table 3). The analysis only included trials where both ET and MC data were available. There was a significant effect of condition, *F*(1,1712) = 16.35, *p* < .001, modified by a significant age-by-condition interaction effect, *F*(1,1712) = 9.63, *p* = .002, and a significant group-by-condition interaction effect, *F*(1,1712) = 4.80, *p* = .03. There was also a significant three-way interaction effect of age, group, and condition, *F*(1,1712) = 4.80, *p* = .02 (lower panel of Fig. 3). The patterns of group differences in age-related changes of % screen looking toward Social and Nonsocial stimuli are largely consistent with the ET findings. Specifically, LL infants exhibited an age-related increase in % screen looking to the Social relative to Nonsocial videos, *B* = 0.34, *SE* = 0.07, *t* = 4.82, 95% *CI*[0.20, 0.48], *p* < .001. However, EL infants did not show developmental changes in % screen looking to the Social relative to Nonsocial stimuli, *p* = .62. LL infants also had greater % screen looking than EL infants specifically for the Social videos across age, *B* = 3.24, *SE* = 1.30, *t* = 2.49, 95% *CI*[0.65, 5.83], *p* = .02. Compared to ET findings, the MC analysis additionally shows that LL infants exhibited an age-related increase in % screen looking specifically to Social videos, *B* = 0.46, *SE* = 0.21, *t* = 2.22, 95% *CI*[0.05, 0.87], *p* = .03.

## Discussion

The overall aim of this study was to assess the utility of using eye tracking to measure social attention in EL and LL infants aged 3–24 months. As in a prior study on toddlers (Venker et al., 2020), we compared looking behavior measures from ET and MC obtained from the same infants. First, infant ET data were collectable and successful calibration was achievable in infants aged between 3 and 24 months for 95.83% of ET sessions. Descriptive statistics showed that compared to MC measures of looking behavior, ET provided lower % screen looking, lower % off-screen looking, and higher % data loss (i.e., lower % valid data). Indeed, model testing revealed that ET yielded significantly reduced % valid data relative to MC. Second, participant-based factors, including age and ASD familial likelihood, did not impact % valid data measured using either ET or MC. Third, there was a significant positive association between ET and MC measures of % screen looking. Lastly, ET and MC yielded comparable findings of age-related change in social attention across LL and EL infants. Both measures revealed that LL infants show a greater % screen looking to Social videos than the EL infants from 3 to 24 months. Furthermore, LL infants exhibited greater attention to social compared to nonsocial information (Social > Nonsocial % screen looking) as they grew older. This developmental emergence of greater attention to social relative to nonsocial information was not found in EL infants. Together, while ET underestimated the duration of looking toward and away from the experimental stimuli compared to MC, the extent of ET data loss did not differ by participant-based factors, and the greater data loss compared to MC did not impact the findings on attention towards social and nonsocial information.

The current study indicated that ET is prone to more data loss, resulting in an underestimation of the duration of attention and inattention. This is consistent with existing evidence obtained from within-subjects ET-MC comparison (Venker et al., 2020). ET determines gaze locations using the locations of corneal reflection, pupil position, and head position (Wass et al., 2013, 2014). When the eye tracker fails to detect these metrics, longer fragments of ET data loss (i.e., flickery data) lead to shorter AOI fixation durations recorded (Wass et al., 2013, 2014). In the current study, bouts of ET data loss when the location of the POG cannot be detected might be caused by periods of looking away from the screen or non-attentional factors, such as infants’ poor head control, head and body movement, fussiness, and droopy and watery eyes (Hessels et al., 2015; Wass et al., 2013, 2014). In contrast, MC relies on a trained human coder to determine the infant’s gaze location. MC is flexible and more robust against data loss due to head position and movements, as coders can annotate looking toward or away from the screen AOI as long as the infants’ eyes are clearly visible (Venker et al., 2020; Wass et al., 2013). Hence, the reduced MC % data loss compared to ET % data loss observed in this study may be due to MC providing more accurate differentiation between inattention and data loss.

While ET is vulnerable to data loss, infant age, and likelihood did not influence the extent of ET data loss in this study. Younger infants and children with ASD may be more likely to exhibit behaviors that can lead to greater data loss, such as head tilting, body rocking, squinting, and non-compliant behaviors (Dalrymple et al., 2018; Venker et al., 2020). Indeed, previous evidence has indicated that ASD phenotypes were related to greater data loss (Wang et al., 2023; Wass et al., 2015). However, the current finding is consistent with ET studies showing that ASD likelihood status did not affect the number of valid trials (Braukmann et al., 2018) and the ability to successfully calibrate in the first two years of life (e.g., Chawarska et al., 2016; Shic et al., 2014), and the amount of data exclusion did not vary by infant age (e.g., Chawarska et al., 2013). It is possible that a greater increase in ET data loss may emerge later in development and for children with a formal ASD diagnosis (Shic et al., 2022). Further investigations are important to determine the association between ET data loss and ASD phenotypes, as the very factors that lead to data loss might serve as a candidate biomarker for ASD (Wang et al., 2023).

Furthermore, we examined the concordance of ET and MC measures of % screen looking. We found a strong positive ET-MC association of % screen looking. Moreover, ET and MC measures show comparable patterns of group differences in developmental changes of attention towards Social versus Nonsocial stimuli indexed by % screen looking. Specifically, EL infants exhibited a reduced % screen looking to the Social stimuli compared to LL infants across ages 3 to 24 months. Moreover, LL, but not EL infants, displayed an age-related increase in their attention to Social versus Nonsocial information. MC is limited in measuring the microstructure of looking behavior due to its reduced spatial and temporal resolutions compared to ET (Aslin, 2007, 2012; Wass et al., 2013). However, we showed high concordance between ET and MC measures of attention. MC reveals comparable group differences between LL and EL infants in social attention patterns as ET when data availability (i.e., an equal number of participants and trials), AOI (i.e., the screen), and attention metric (i.e., % screen looking) are equalized. Our supplementary analysis (see SI) that used all available data from ET and MC also revealed comparable results between the two measures. While ET provided fewer data at both the visit level (failure to calibrate) and trial level (greater % data loss), ET data loss did not impact the overall experimental findings. For studies that aim to measure looking duration toward large AOIs in participants vulnerable to ET data loss, MC can provide a viable alternative measurement tool or be used as an additional measurement for validating ET results (Venker & Kover, 2015; Venker et al., 2020; Wass et al., 2013).

Reduced attention to social information in ELs may lead to a cascade of impairments in social communication during development (Bradshaw et al., 2022; Mundy & Bullen, 2022). We found that LL infants showed increased attention to social compared to nonsocial stimuli during the first two years of life, consistent with existing studies using ET (Frank et al., 2009, 2012) and looking behavior measures (Bahrick et al., 2016). Increased social attention supports learning and is related to the development of broader social communication skills (Salley & Colombo, 2016). In contrast, EL infants showed reduced social attention compared to LL infants across the first 2 years of life. This finding aligns with existing evidence indicating that EL infants later diagnosed with ASD exhibited disrupted social attention (Chawarska et al., 2013; Jones et al., 2016; Jones & Klin, 2013). EL infants did not show a higher % screen looking at the Nonsocial than Social videos. We cannot infer social or nonsocial preference in our sample, as we did not use a preferential looking paradigm that displays social and nonsocial stimuli side-by-side (Pierce et al., 2011, 2016; Wen et al., 2022). Early-emerging differences in social attention change the information that children take in from the environment and may further restrict opportunities for social learning, contributing to impairments in social learning, social communication, and executive functions observed in children with ASD (Bradshaw et al., 2022; Brandes-Aitken et al., 2020; Klin et al., 2015; Mundy & Bullen, 2022).

The current findings underscore the potential utility of ET measures of attention as a risk marker of ASD likelihood in early development. Reduced attention to social information has been identified as a viable ET biomarker for ASD in toddlers (Jones et al., 2023) and older children (Shic et al., 2022), and this study provides more evidence that ET may offer an avenue for ASD detection early in infancy, despite higher data loss. Studying social attention in young infant siblings of children with a confirmed ASD diagnosis facilitates the detection of early risk markers and attention functions associated with broader autism phenotype (Elsabbagh & Johnson, 2010; Rogers, 2009) before an ASD diagnosis can be made around three years of age. Recently, Jones et al. (2023) showed that an ET measure of social attention in 16- to 30-month-olds predicted ASD diagnosis by clinical experts with satisfactory sensitivity and specificity.

While the present study supports ET as a promising tool for the early identification of attentional risk markers for ASD likelihood, our findings need to be interpreted in light of several limitations. First, the study is limited by a small sample size, especially for the EL group. This may have limited the power of detecting any age-related change of % screen looking at the Social versus Nonsocial videos in the EL infants. Moreover, EL infants present a heterogeneous group, half of whom will later be diagnosed with ASD (~ 20%) and manifest subclinical features of ASD (~ 30%). Due to the small sample size, this study was not powered to compare diagnostic outcome groups and findings here are limited to predicting ASD likelihood. Further analyses with a larger sample are needed to determine whether reduced social attention differentiates between LL children, EL children with and without ASD diagnosis and comment on the utility of social attention as an ASD biomarker in early infancy. Second, we used a relatively large AOI (i.e., the entire screen) in order to perform comparisons between ET and MC results. This might have reduced the specificity of atypical attentional patterns that differentiate EL from LL infants. Lastly, our examination of participant-specific characteristics was limited to age and ASD likelihood. Further investigation of other factors like movement, eye color, eye blinks, and seating position can extend these findings and provide a comprehensive characterization of factors that impair ET data quality in EL and LL infants (Hessels et al., 2015; Wang et al., 2023; Wass et al., 2014).

In conclusion, early identification of atypical social attention patterns associated with elevated likelihood for ASD is critically important for early detection and targeted interventions. Our results demonstrated that ET data can be obtained in LL and EL infants aged 3 to 24 months with a high success rate. ET yielded a lower proportion of valid data for determining attention to the screen compared to MC. However, the proportion of valid ET data obtained did not vary by infant age or ASD likelihood and there was a high concordance between ET and MC measures of attention. Importantly, ET revealed comparable patterns of experimental effects as MC, albeit with a reduced proportion of valid data. Specifically, LL infants displayed: (1) greater attention towards social information than EL infants, and (2) greater attention for Social over Nonsocial videos that increased with age during the first two years. Hence, ET remains a promising tool for identifying attention risk markers associated with ASD likelihood and broader autism phenotype in early development, before reliable ASD diagnoses can be made.
