# Supplementary material for: Infant Social Attention Associated with Elevated Likelihood for Autism Spectrum Disorder: A Multi-Method Comparison
Source: J Autism Dev Disord. Author manuscript; Available in PMC 2025 Jul 1. (PMC11911713; doi:10.1007/s10803-024-06360-z)
Supplement: Supplementary file 1 [file NIHMS2032509-supplement-Supplemental.docx]

AgSupplemental Information

**The Effect of Age, Group, and Task Condition on % Screen Looking measured using ET and MC**

With the available ET data, the LME model revealed a significant effect of condition, *F*(1,1712)=15.92, *p*<.001, and a significant group-by-condition effect, *F*(1,1721)=16.40, *p*<.001, which was modified by a marginally significant group-by-condition interaction effect, *F*(1,1721)=3.70, *p*=.05 and a significant three-way interaction between age, group, and condition, *F*(1,1721)=7.98, *p*=.005. The upper panel of SI Figure 1 displays the interaction effect on the ET measure of % screen looking. The LL infants displayed greater % screen looking toward the Social relative to the Nonsocial videos as they grew older, *B*=0.32, *SE*=0.08, *t*=3.91, 95% *CI*[0.16, 0.48], *p*<.001. In contrast, the EL infants did not show developmental changes in attention to the Social versus Nonsocial videos, *p*=.44. The group difference was also manifested in the higher % screen looking in the LL than EL infants for the Social stimuli across ages, *B*=3.86, *SE*=1.75, *t*=2.20, 95% *CI*[0.37, 7.35], *p*=.03. Moreover, the LL infants showed marginally age-related increase in % screen looking to the Social video, *B*=0.45, *SE*=0.24, *t*=1.90, 95% *CI*[-0.02, 0.93], *p*=.06.

For the %screen looking estimated from MC, there was a significant effect of condition, *F*(1,1787)= 8.78, *p*=.003, modified by a significant age-by-condition interaction effect, *F*(1,1787)=14.09, *p*<.001, and a significant group and condition interaction effect, *F*(1,1787)= 6.86, *p*=.01. There was also a significant three-way interaction effect of age, group, and condition, *F*(1,1787)=3.92, *p*=.048 (lower panel of SI Figure 1). The patterns of group differences in age-related changes of % screen looking toward Social and Nonsocial stimuli are largely consistent with the ET findings. Specifically, the LL infants exhibited age-related increase in % screen looking to the Social relative to Non-social videos, *B*=0.37, *SE*=0.07, *t*=5.14, 95% *CI*[0.23, 0.51], *p*<.001. However, the EL infants did not show developmental changes in % screen looking to the Social relative to Nonsocial stimuli, *p*=.29. The LL infants also had greater % screen looking than EL infants specifically for the Social videos across ages, *B*=3.07, *SE*=1.30, *t*=2.36, 95% *CI*[0.48, 5.66], *p*=.02. Additionally, LL infants exhibited age-related increase in % screen looking specifically to the Social videos, *B*=0.60, *SE*=0.21, *t*=2.85, 95% *CI*[0.18, 1.01], *p*=.01.

**SI Fig 1**. The interaction effect of age, autism risk status (i.e., group: low likelihood versus elevated likelihood) and condition (Social versus Non-social) on the proportion of time looking at the screen over the stimulus presentation duration, separately for the eye tracking (ET) and manual coding (MC) measures. All trials with available ET or MC data were included in model testing. Age was mean-centered for model fitting (*M*=11.4). 

*Notes*: +*p*=.06, **p*<.05; LL=low likelihood; EL = elevated likelihood.
